# Functional analysis of a novel parasitic nematode-specific protein of *Setaria digitata* larvae in *Culex quinquefasciatus* by siRNA mediated RNA interference

**DOI:** 10.1186/s13071-018-3096-x

**Published:** 2018-10-10

**Authors:** Magalla Bastian Chalitha Lakmal Somarathne, Yasanthi Illika Nilmini Silva Gunawardene, Naduvilath Vishvanath Chandrasekharan, Arjuna Nisantha Bandara Ellepola, Ranil Samantha Dassanayake

**Affiliations:** 1 Department of Chemistry, Faculty of Science, University of Colombo, Colombo 03, 00300 Sri Lanka; 2 Molecular Medicine Unit, Faculty of Medicine, University of Kelaniya, Ragama, 11010 Sri Lanka; 3 Department of Bioclinical Sciences, Faculty of Dentistry, Health Sciences Center, University of Kuwait, Kuwait City, Kuwait

**Keywords:** *Setaria digitata*, *SDNP*, RNAi, *Culex quinquefasciatus*, Gene knockdown

## Abstract

**Background:**

Functional analysis of animal parasitic nematode genes is often quite challenging due to the unavailability of standardised *in vitro* culture conditions and lack of adequate tools to manipulate these genes. Therefore, this study was undertaken to investigate the suitability of *Culex quinquefasciatus*, as an *in vivo* culture platform for *Setaria digitata* larvae and RNA interference (RNAi), as a post-transcriptional gene silencing tool to study the roles of a vital gene that encodes a novel parasitic nematode-specific protein (*SDNP*).

**Results:**

The red colour fluorescence detected following RNAi injection to the thorax of *C. quinquefasciatus* indicated the uptake of dsRNA by *S. digitata* larvae. The reduction of *SDNP* transcripts in siRNA treated larvae compared to non-treated larvae, as determined by qPCR, indicated that the siRNA pathway is operational in *S. digitata* larvae. The observation of motility reductions and deformities during the development indicated the association of *SDNP* in larvae locomotion and development processes, respectively. The irregularities in the migration of larvae in mosquitoes and elevated survival rates of mosquitoes compared to their untreated counterparts indicated reduced parasitism of *S. digitata* larvae in mosquitoes upon targeted downregulation of *SDNP* by siRNA treatment.

**Conclusion:**

SDNP plays vital roles in muscle contraction, locomotion, development processes, larval development and parasitism of *S. digitata*. Its ubiquitous presence in parasitic nematodes and its absence in their hosts provide a tantalising prospect of the possibility of targeting SDNP for future development of anthelmintic drugs. The susceptibility of the larval stages of *S. digitata* for RNAi in *Culex quinquefasciatus* was also demonstrated for the first time in this study.

## Background

Nematodes are the most abundant metazoans, capable of inhabiting both terrestrial and aquatic habitats. This facilitates their ability to cause both human and animal health problems, as well as to impair crop production worldwide. Gastrointestinal nematodes infect 3.5 billion humans annually worldwide, and cause around 125,000 deaths per year, whereas filarial nematodes infect 120 million people in the globe annually, disabling around 40 million of them [1–3]. Additionally, plant-parasitic nematodes destroy approximately 12.3% of the annual crop yield, and animal parasitic nematodes kill millions of livestock animals. This contributes to a loss of billions of US dollars annually and necessitates the allocation of additional billions of US dollars for anthelminthic drugs for livestock [4, 5]. Strategies for controlling this problem have mainly focused on chemical treatments [5]. However, due to the limited efficiency and high toxicity of the available drugs, adverse effects on the environment, and emergence of drug resistance in parasites, these chemical treatment methods have become somewhat ineffective [6–8]. Hence, global interest has recently arisen in developing more effective controlling strategies for parasitic nematodes. In this context, screening of the nematode genome will enable us to gain a comprehensive knowledge of the molecular mechanisms associated with the development of the nematode, which may facilitate the control of parasitic nematodes [9, 10].

RNA mediated gene silencing has become a promising and unbiased tool in genetic screening [11]. RNA interference (RNAi) is a double-stranded RNA (dsRNA) induced post-transcriptional, homology-dependent gene silencing mechanism, naturally occurring in organisms, assumed to be involved in defence against virus attacks, heterochromatinisation and retrotransposon silencing [11–13]. RNAi was initially utilised in the genetic screening of *Caenorhabditis elegans*, and after that, this technique was expanded to other invertebrates (nematodes, insects, molluscs), vertebrates (fish, mice, humans), plants, fungi and algae [14–17]. RNAi allows the temporary shut-down of gene expression more specifically and rapidly and gives an insight into gene function [18, 19].

*Setaria digitata* is a parasitic nematode that naturally resides in the peritoneum and mesentery of cattle, zebu and water buffalo. Under normal circumstances, the nematode does not show any pathogenicity towards the natural hosts. The microfilariae produced by the adult female worm travel into the bloodstream of the cattle and are transmitted into new hosts *via* mosquito bites. However, when the larvae are transfected into non-permissive hosts such as goats, sheep and horses, they invade the brain, spinal cord, and eyes, causing cerebrospinal setariosis, conjunctivitis, and filariasis oculi, respectively [20]. Intriguingly, studies on *S. digitata* have revealed that it has a high resemblance to the human parasitic nematode *Wuchereria bancrofti* with regard to its morphology, antigenicity and histology [21, 22]. Hence *S. digitata* has been recommended as a model organism for studying *W. bancrofti* and *Brugia malayi* [23]*.*

Unveiling the biology of the parasitic nematodes genes, which are specific and functionally crucial for their survival, is crucial in identifying targets to control the nematode population [16]. *SDNP* is such a gene found in *S. digitata*. Intriguingly, this gene has demonstrated high sequence similarities with putative proteins of *W. bancrofti* (79%), *Brugia malayi* (77%) and *Loa loa* (81%). Moreover, bioinformatics studies of *SDNP* revealed that this protein contains a nuclear localisation signal, an RNAP_Rpb7_N_like domain, and an inter-domain linker of muscle-specific twitchin kinase similar to that of *C. elegans* [24]. Considering the background, our study focused on the functional characterisation of *SDNP*.

Commonly, RNAi protocols are practised by isolation of the nematodes from their natural hosts and maintaining them in artificial liquid cultures (*in vitro*). These *in vitro* protocols reduce the efficiency of RNAi due to several shortcomings such as difficulty in maintaining the worms’ viability in the culture, poor RNAi trigger delivery, and compatibility issues of the culture medium with different life stages of the worm [25]. To overcome these drawbacks of *in vitro* protocols, in our study an *in vivo* approach was developed, where *S. digitata* larval stages were cultured in its intermediate host, *Culex quinquefasciatus*, and the RNAi trigger (siRNA) was directly injected into the mosquito [19], and the possibility of using the mosquito as a better siRNA delivery platform examined. Furthermore, we focused on developing an effective RNAi protocol to specifically shut down the gene of interest to study its function and explore the gene’s potential as a likely drug target.

## Methods

### Mosquito colony establishment

*Culex quinquefasciatus* were collected from a cattle trap and maintained at 27 °C with 80% relative humidity, with cycles of 16 h of light and 8 h of darkness, in 12 × 12 × 12-inch cages [26]. The eggs obtained from these mosquitoes were hatched in cups containing pre-boiled water, and the larvae which emerged from the eggs were fed with a larval diet (brewer yeast, bovine liver powder, tuna meal) until pupae appeared. The pupae were then separated from the larvae and placed in containers with fresh water in separate cages for 36 h until adult mosquitoes emerged. These were then fed with 10% sucrose solution [27]. They were used as an *in vivo* culturing medium for *S. digitata* microfilariae.

### Mosquito infection with *S. digitata*

Adult female worms obtained from a cattle slaughterhouse in Welisara, Ragama, Sri Lanka were cut into small pieces after washing three times with mosquito physiological saline (MPS) [28] to release microfilariae (MF) into a tube containing 300 μl MPS. This was vortexed and centrifuged at 1500× *rpm* for 30 s, and the supernatant recovered was then re-centrifuged at 1500× *rpm* for 3 min to obtain a pellet containing MF, which was then re-suspended in MPS to achieve a microfilariae (MF) concentration of 40 MF/μl.

The thorax of anaesthetized adult mosquitoes (aged 3 days) on ice [29] was injected with a volume of 0.5 μl of microfilariae suspension using a pre-pulled borosilicate glass capillary attached to a manual injector [29–31]. The injected mosquitoes were kept in paper cups covered with a net while providing them with cotton pads soaked with the sucrose solution.

### siRNA synthesis, Cy3 labelling and RNAi treatment

The entire coding sequence of *SDNP* was divided into four overlapping regions*,* and each of them was amplified by separate PCR reactions using four separate primer sets containing T7 promoter sequence at 5' ends (Table 1) to enrich the total yield of the template and PCR products of overlapping regions were used to synthesize siRNA. The PCR conditions used for the amplification were initial denaturation at 94 °C for 2 min, followed by 40 cycles of denaturation at 94 °C for 30 s, annealing at 55 °C for the primer sets SDNP-1-F/R, SDNP-2-F/R and SDNP-3-F/R and 45 °C for the primer set SDNP-4-F/R, and extension at 72 °C for 30 s with a final extension of 10 min at 72 °C (Table 1).

**Table 1:** Primer sets used for the synthesis of dsRNA. The positions of the primers with respect to *SDNP* sequence (GenBank: GU222920), sequence corresponding to T7 promoter region of primers (underlined), the size of the PCR products, and the optimized annealing temperatures of primer sets are given

Primer	Position	Sequence (5'-3')	Product size (bp)	Annealing T (°C)
SDNP-1-F	1	TAATACGACTCACTATAGGGCGACGAGGGTTCCATTGAGTGA	154	55
SDNP-1-R	134	TAATACGACTCACTATAGGGGCTTCCTGACAAGCCAAACAT		
SDNP-2-F	155	TAATACGACTCACTATAGGGCGACGAGGGTTCCATTGAGTGA	150	55
SDNP-2-R	282	TAATACGACTCACTATAGGGTTGGAATATAAACATGCGGTATA		
SDNP-3-F	305	TAATACGACTCACTATAGGGCTACCGGTATCAGAACTCAGAA	157	55
SDNP-3-R	462	TAATACGACTCACTATAGGGTAGCGCTGGACCGAATTCTTTT		
SDNP-4-F	463	TAATACGACTCACTATAGGGGGAAATGGAACTTGTGAAATAA	155	45
SDNP-4-R	618	TAATACGACTCACTATAGGGTCAGTAATTAATCAAATT		

*Abbreviation*: *T* temperature

The resulting PCR fragments were transcribed separately *in vitro* by T7 RNA polymerase (New England Biolabs, Hitchin, UK) using slightly modified manufacturer’s protocols, to yield a high amount of dsRNA. Briefly, the *in vitro* transcription reaction was carried out overnight at 37 °C in a 120 μl reaction mix containing 81.5 μl of nuclease-free H_2_O, 12.0 μl of 10× T7 buffer, 3.0 μl of DTT (100 mM), 10 μl (2 μg) of PCR product, 12 μl of rNTP mix (80 mM), 1.5 μl of T7 RNA polymerase (18 U/μl). The RNA products of *in vitro* transcribed DNA were first treated with DNase 1 (New England BioLabs) to remove DNA templates and then incubated at 75 °C for 5 min before cooling them down to room temperature for the complementary strands of synthesized RNA to anneal to make dsRNA. The dsRNA (assessed by gel electrophoresis) was digested with RNase III (New England Biolabs) according to the manufacturer’s protocol to produce 21–23 mer siRNA. They were analyzed by polyacrylamide gel electrophoresis before labelling with Cy3 fluorescent dye using a Silencer® siRNA Labeling Kit - Cy™3 dye (Ambion, Life Technologies, California, USA) according to the manufacturer’s instructions [32].

The ds-RNA synthesized from the gene encoding for the green fluorescent protein (GFP) of *Aequorea victoria* was also used as the non-specific siRNA control as this gene neither has any homologous sequences in *S. digitata* nor mosquito. pCRII TOPO vector (pCRII TOPO-*GFP*) containing *GFP* was used in the synthesis of GFP dsRNA. The vector was digested with *Kpn* I [7.5 μl nuclease free H_2_O, 2.0 μl 10× buffer, 0.5 μl *Kpn* I (15 U/μl)*,* 10 μl DNA (200 ng/μl); incubated 1 h at 37 °C] and *Xho* I [10 μl nuclease free H_2_O, 3.0 μl 10× buffer, 0.3 μl BSA, 0.5 μl *Xho* I (20 U/μl), 16.2 μl DNA (200 ng/μl); incubated 3 h at 37 °C] separately. The *Kpn* I digested vector was *in vitro* transcribed by T7 RNA polymerase using the same protocol indicated above. The *Xho* I digested vector was *in-vitro* transcribed by Sp6 RNA polymerase using the following reaction; 60 μl of nuclease-free H_2_O, 8.0 μl of 10x Sp6 buffer, 2 μg (10 μl) of pCR II-TOPO vector, 8 μl of rNTP mix (80 mM), 2 μl of Sp6 RNA polymerase (20 U/μl), incubated at 40°C for 4 h. The separately *in vitro* transcribed RNA was mixed and incubated at 75 °C for 5 min and cooled down to room temperature for the complementary strands of synthesised RNA to anneal to make dsRNA.

The *S. digitata* infected mosquitoes were initially injected with 150 ng of Cy3 labelled *SDNP* siRNA mix into their thorax at 12 h post infection (hpi) to monitor the uptake of siRNA by larvae. To target the L2 to L3 larvae transformation, non-labelled siRNA mix was injected at eight days post infection (dpi). The *GFP* siRNA (150 ng) was injected as a negative control.

### Mosquito dissection

The mosquitoes infected with *S. digitata* and injected with *SDNP* siRNA, non-injected, *GFP* siRNA-injected and buffer-injected were dissected at different time intervals (24 hpi, 8–14 dpi) under a dissection microscope. In this process, the bodies of cold anaesthetized mosquitoes were separated into the thorax, midgut, and head, and each of these body parts was further dissected to release the larvae on to glass slides. The MF obtained from the smeared content on the slides at 24 hpi were visualized under the fluorescent microscope using Cy3 filter set (excitation: 550 nm, emission: 570 nm) to detect the uptake of siRNA. The transformation of L2 larvae to L3 was recorded from the mosquitoes dissected at 8 to 14 dpi, and the following data were recorded only from the mosquitoes dissected at 14 dpi: (i) larvae length and width (Image J software was utilized in obtaining the measurements); (ii) larvae location in the mosquito (larvae in the head, thorax and abdomen of the mosquito; (iii) larvae motility (motility of the RNAi treated and control worms were compared according to a previously used pre-fashioned 5-point scheme based on Song et al. [19]). The scoring scheme was as follows: 1, immobile, dead; 2, compromised motility; 3, partial movement of the body; 4, slightly reduced motion in the body; and 5, all parts of the body are vigorously twisting. Values in between 5 and 1 in the scale were assigned according to the decreasing motility blindly by an independent evaluator; (iv) at 14 dpi, mosquito survival rate was also measured by counting the live mosquitoes.

### Relative quantification of SDNP mRNA level

Total RNA was extracted at two dpi and 14 dpi from non-injected, *GFP* siRNA injected, *SDNP* siRNA-injected and buffer-injected mosquitoes infected with S*. digitata* using a GenoSpin R^TM^ Total RNA Extraction Kit (CEYGEN Biotech, Colombo, Sri Lanka) according to the manufacturer’s instructions. Five mosquitoes were used from each batch. DNA contamination in extracted RNA was removed by treating with DNase I and heat inactivating the DNase I at 75 °C for 5 min. cDNA synthesis was carried out using RNA (2 μg) purified using an RNeasy Plus Mini Kit (Qiagen, California, USA) in a 20 μl reaction mix containing 5.0 μl of M-MLV transcription buffer, 1.0 μl of oligo dT primers (5 nM), 1.25 μl of dNTP mix (10 nM), 1.0 μl RNase inhibitor (20 U), 1.0 μl M-MLV reverse transcriptase (200 U, Promega, California, USA), RNA (2 μg), and RNase free water according to the vendor’s protocol.

Relative quantification of *SDNP* mRNA levels was carried out by quantitative real-time PCR [CFX96 RealTime PCR System (Bio-Rad)] using SYBR green as the DNA binding dye. qPCR conditions were initial denaturation at 95 °C for 2 min, followed by 40 cycles of denaturation at 95 °C for 30 s annealing at 60 °C for SDNP primers and 55 °C for actin primers for 45 s (Table 2), and with an extension at 72 °C for 30 s. In qPCR, the actin gene (GenBank: AF079359.1) was used as the internal amplification control in a separate tube for each qPCR reaction. In calculating the relative mRNA expression level, the 2^-∆∆CT^ method was used as mentioned in qPCR Application Guide (BioRad). The data obtained were presented as the mean ± standard deviation (SD). A *P* value < 0.05 was considered statistically significant.

**Table 2:** Primers used for qPCR. The primer sequences of the target (SDNP) and internal control (Actin) are given

Primer	Sequence (5'-3')	Position	Product size (bp)	Annealing T (°C)
SDNP amplification				
SDNP-RTPCR-FP	AAGACTTGCAACTCCATCTCGAT	219	170	60
SDNP-RTPCR-RP	ATTTTCTCGCTGACCACCACAACT	366		
*S. digitata* actin amplification (reference gene)				
SDACT-RTPCR-FP	CGCTCGAGAAATCTTACGAATTGC	707	147	55
SDACT-RTPCR-RP	TCCTTTCTGATATCGATATCACATT	854		

*Abbreviation*: *T* temperature

## Results and discussion

### siRNA uptake by *S. digitata* microfilariae in the mosquito host

**Fig. 1:**
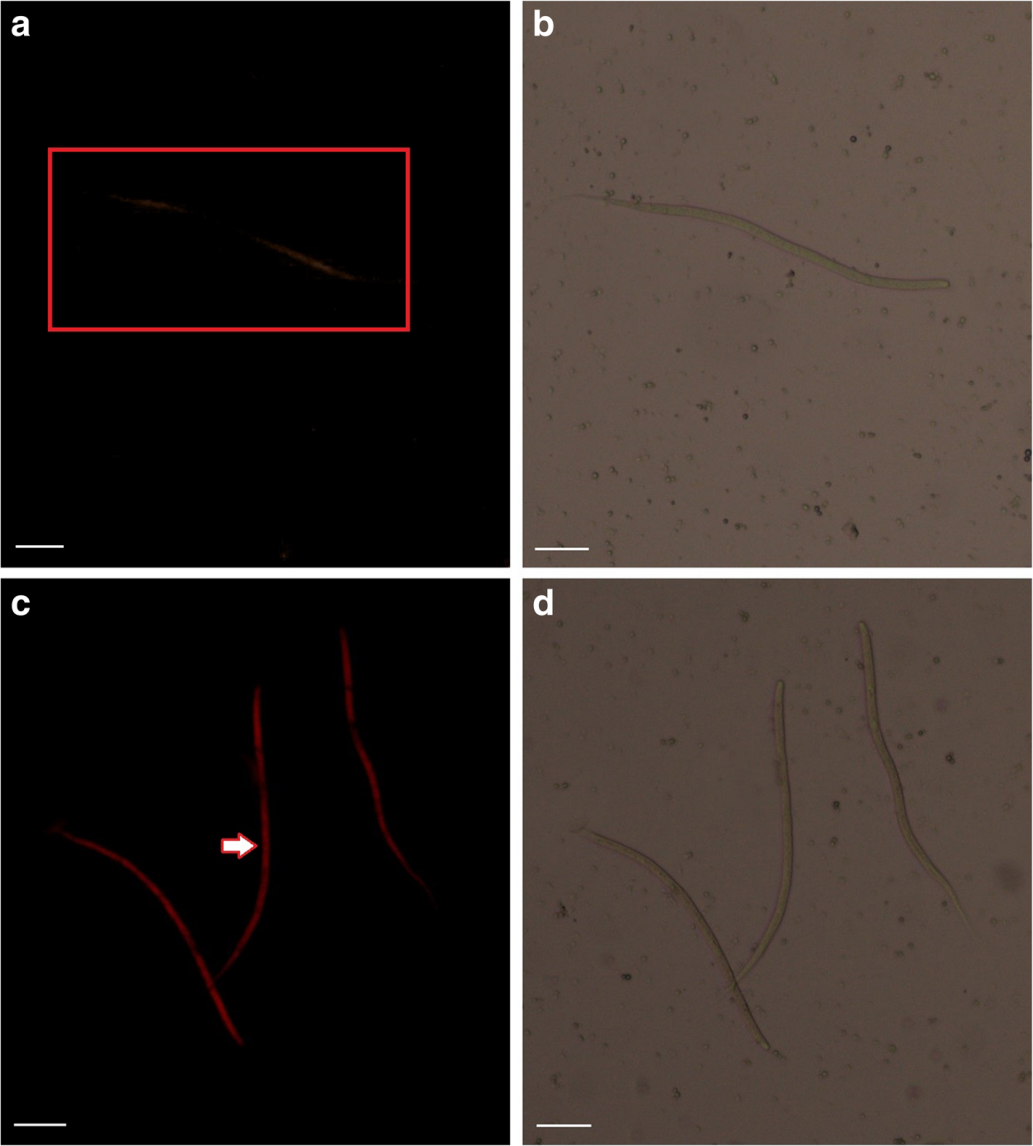
Uptake and distribution of Cy3 labelled *SDNP* siRNA by microfilariae at 24 hpi. **a** Fluorescent image of microfilariae from siRNA non-injected mosquitoes (position indicated by a red square). **b** Bright field image of microfilariae from siRNA non-injected mosquitoes. **c** Fluorescent image of microfilariae from Cy3 labelled *SDNP* siRNA-injected mosquitoes (arrow indicates the dispersion of Cy3 labelled siRNA). **d** Bright field image of microfilariae from Cy3 labelled *SDNP* siRNA-injected mosquitoes. Fluorescence was visualised using Olympus BX53 using Cy3 filter. Magnification 400×. *Scale-bars:* **a-d**, 20 μm

A previous study demonstrated the possibility of *in vivo* suppression of the cathepsin L-like cysteine protease gene of *B*. *malayi* using RNAi in *Aedes aegypti* [19]. Therefore, in this study, the possibility of using *C. quinquefasciatus* as an *in vivo* siRNA delivery platform for developing *S. digitata* larvae was tested, where the transformation of MF to L3 larvae of the latter organism has been observed. Since the previously stated study revealed the dissemination of siRNA to the MF of *B. malayi* through the mosquito, in the current study the uptake of the Cy3 labeled RNAi trigger by MF was investigated by, comparing the internal fluorescence of the MF with the mosquito physiological saline (MPS) injected control under a fluorescent microscope (Fig. 1a). A dispersion of the fluorescent signal was observed throughout the body of the tested MF at 24 hpi (Fig. 1c) indicating that uptake of Cy3 labelled siRNA by MF is occurring in the mosquitoes. Although the mechanism of siRNA uptake is unclear at this point, it is possible that this would occur *via* feeding or through the cuticle of MF. Therefore, here, it was possible to recreate the *in vitro* siRNA soaking conditions for *S. digitata* larvae *in vivo*, and the possibility of utilizing the mosquito as a siRNA delivering platform for parasitic nematodes hosted by the mosquito was affirmed.

### The effect of siRNA on SDNP mRNA level of MF

**Fig. 2:**
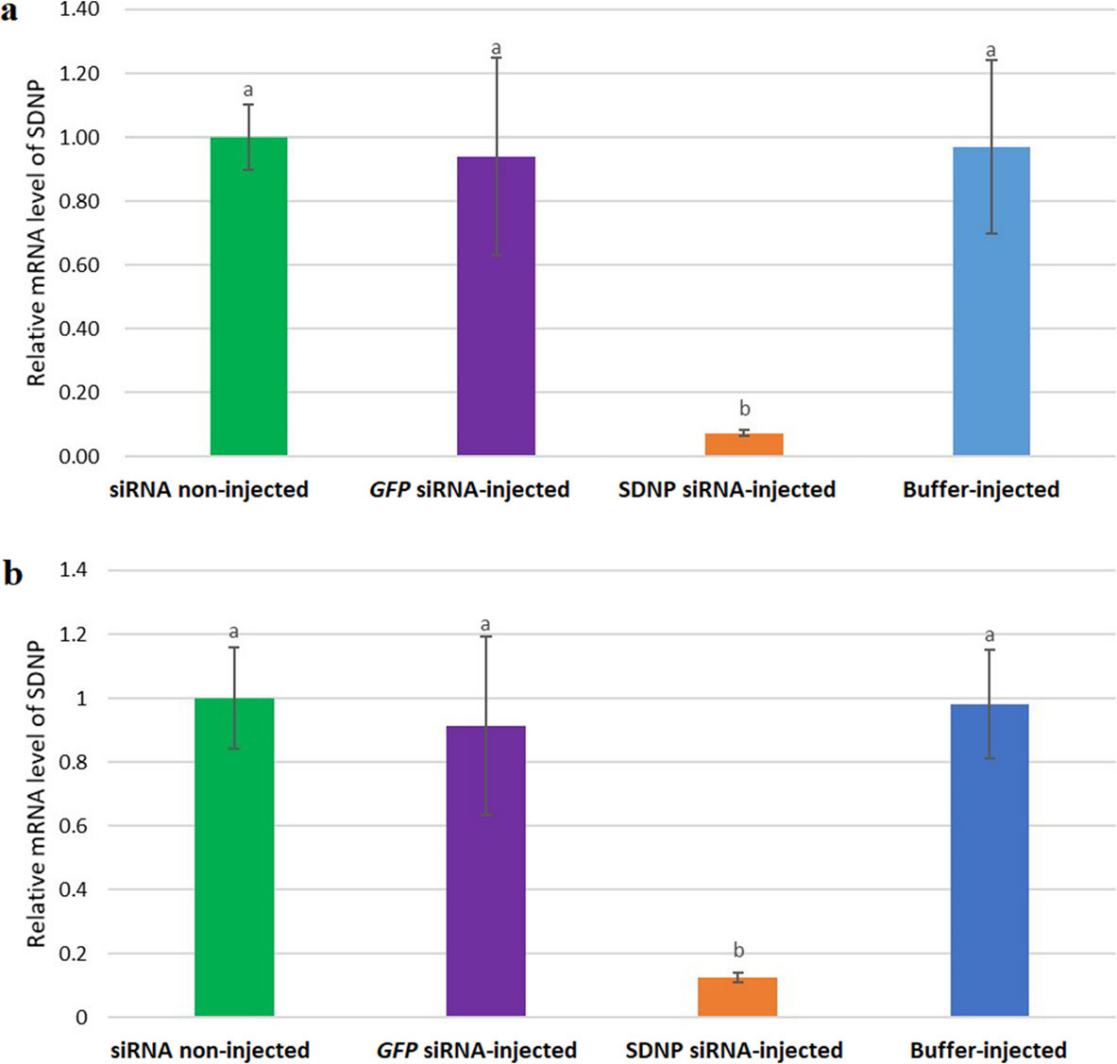
Relative mRNA expression changes owing to the RNAi. **a** Relative mRNA levels of *SDNP* at two dpi. **b** Relative mRNA levels of *SDNP* at 14 dpi. Changes of *SDNP* specific transcript levels were analysed by qPCR, and the 2^-∆∆CT^ method was used in evaluating the results. For the reference gene, *S. digitata* actin gene was used. *GFP* was used as the non-specific control. *SDNP* transcript level was presented as relative to that of non-treated worms. Each experiment was performed in triplicate. Bars display mean ± SD. Statistical analysis was performed using Student’s t-test (*P <* 0.05). Significant differences are represented by different lowercase letters

Several recent studies involving RNAi on nematodes showed that genes could be specifically suppressed by soaking the nematode in *in vitro* culture media consisting of RNAi triggers [25, 32–35]. In this study, instead of using the *in vitro* culture conditions, we recreated the soaking conditions *in vivo* by injecting the mosquitoes harbouring nematode larvae with siRNA. The RNAi effect on *SDNP* transcript level was assessed two dpi, and 14 dpi of siRNA using qPCR and the knockdown of the gene was determined by comparing the transcript levels of the worms derived from non-injected, buffer-injected and *GFP* siRNA-injected mosquitoes. A significant suppression of the target gene expression was observed in this study, and the transcript abundance of the *SDNP* was reduced by 93% (unpaired two-tailed t-test, *t*_44_ *=* 15.8716, *P* = 0.05) and 87.4 % (unpaired two-tailed t-test, *t*_44_ *=* 15.2220, *P* = 0.05) in the worms from *SDNP* siRNA-injected mosquitoes, compared to the non-injected at 2 dpi and 14 dpi, respectively, compared to worms from non-injected mosquitoes (Fig. 2a, b). Worms from the buffer-injected, and *GFP* siRNA-injected mosquitoes displayed no significant changes in the *SDNP* transcript levels (Fig. 2a, b). The qPCR results obtained proved the worms’ susceptibility to specific RNAi while showing the effectiveness of the new siRNA delivery platform developed in this study and the capability of siRNA to knockdown the targeted gene specifically in this parasitic nematode.

### Changes in developmental phenotypes and motility of larvae because of RNAi

**Fig. 3:**
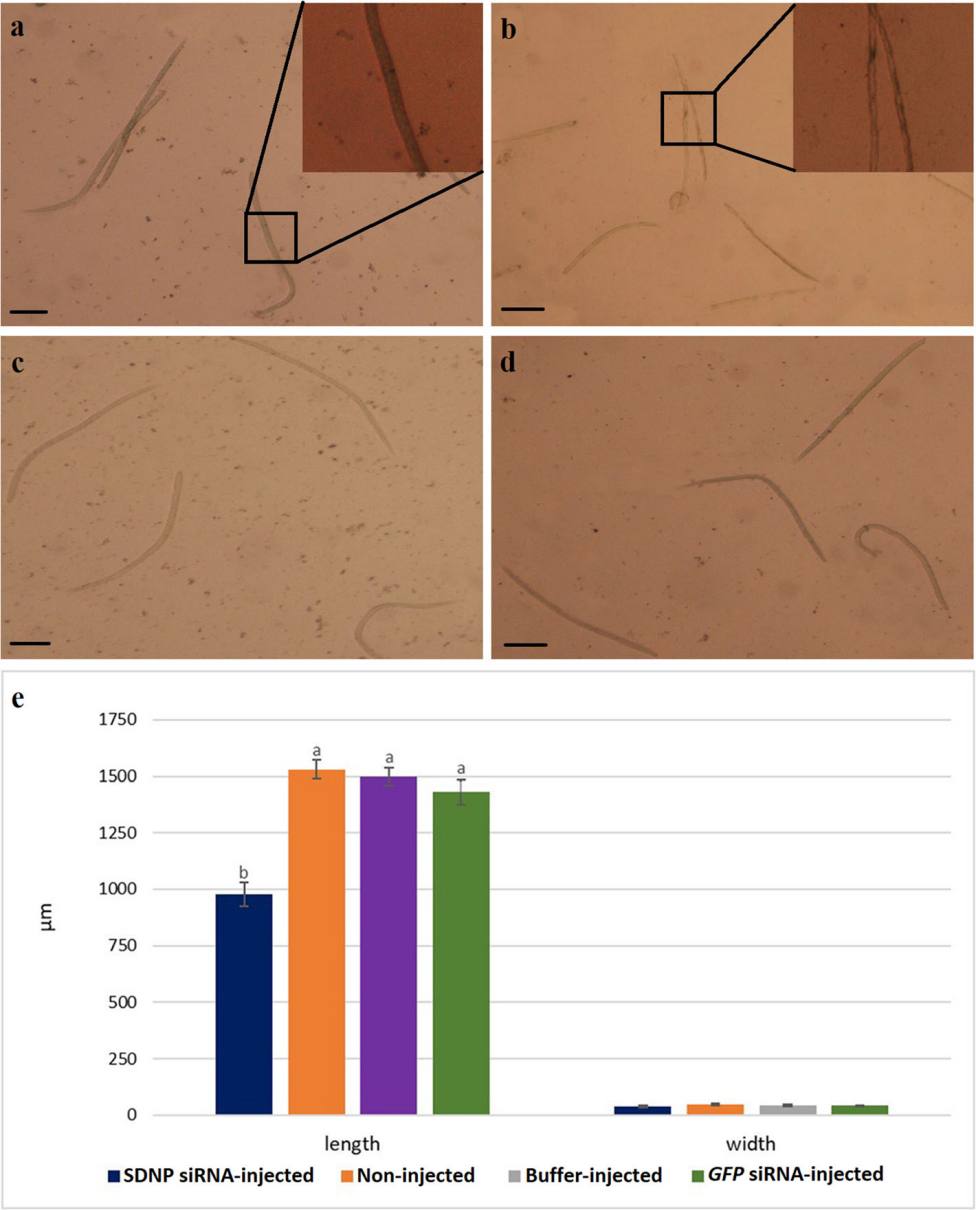
Larvae development in *C. quinquefasciatus* mosquitoes at 14 dpi. **a** Larvae in siRNA non-injected mosquito. **b** Larvae in *SDNP* siRNA-injected mosquito. **c** Larvae in *GFP* siRNA-injected mosquito. **d** Larvae in buffer-injected mosquito (magnification 400×). **e** Mean length and width of larvae form *SDNP* siRNA-injected, non-injected, buffer-injected and *GFP* siRNA-injected mosquitoes. To target L2 to L3, siRNA injections were made at 8 dpi. Triplicates were performed for each experiment. Bars represent mean ± SD. Significant differences at *P* < 0.05 are represented by different lowercase letters. *Scale-bars*: **a**-**d**, 400 μm

**Fig. 4:**
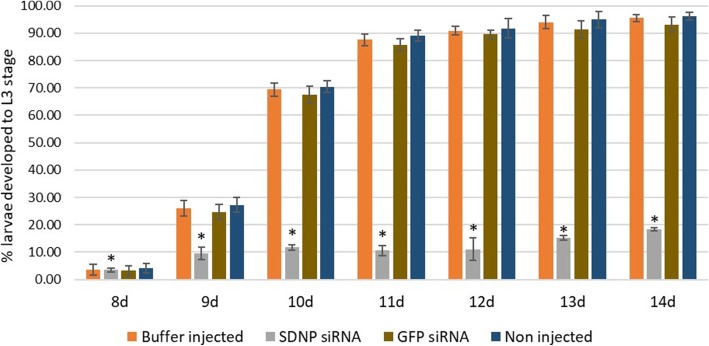
Percentage of larval development to L3 stage out of total larvae inside the mosquito. *S. digitata* larvae from non-injected, *SDNP* siRNA-injected, *GFP* siRNA-injected and buffer-injected *C. quinquefasciatus* are represented by four different patterns in the graph. To target L2 to L3, siRNA injections were made at 8 dpi. Triplicates were performed for each experiment. Bars represent mean ± SD. Significant differences at *P* < 0.05 are indicated by *

To study the phenotypic changes, if any, associated with the transformation of L2 to L3 in the *SDNP* siRNA-injected group in comparison to the control mosquito groups, the infected mosquitoes were dissected to recover the larval stages at eight dpi to 14 dpi following the RNAi trigger. A significant reduction in body length and width together with deformities in the body structure was observed in the larvae from *SDNP* siRNA-injected mosquitoes at 14 dpi (Fig. 3b, e) compared to the non- injected, buffer-injected and *GFP* siRNA-injected counterparts (Fig. 3a, c-e). In addition, aberrations, with larval lethality was also observed in *SDNP* siRNA treated larvae compared to its control groups, and with ~96% of L2 to L3 transformation in non-treated, ~95% in buffer-treated and ~93% in *GFP* siRNA treated compared to ~18% of such transformation in treated at 14 dpi (Fig. 4). The L2 and L3 larvae were differentiated based on their length of the body. No significant developmental changes were observed in the worms from *GFP* siRNA-injected mosquitoes suggesting the phenotypic changes are specific to *SDNP* siRNA and not due to the injection of non-specific siRNA. Therefore, it is reasonable to assume these phenotypic changes are most likely due to the downregulation of *SDNP* transcripts, which in turn affects the larval development. The previous finding reported by us, i.e., the ubiquitous expression of *SDNP* in all life stages of *S. digitata* and also its predominant expression in longitudinal muscle, reproductive systems, and developing embryos [24, 36], together with the findings of the present study, i.e. association of *SDNP* downregulation with irregularities in the larvae development, transformation arrest, changes in phenotypes, etc. in larvae can be used to further strengthen our previous suggestion regarding the involvement of *SDNP* in growth and development of nematodes.

**Fig. 5:**
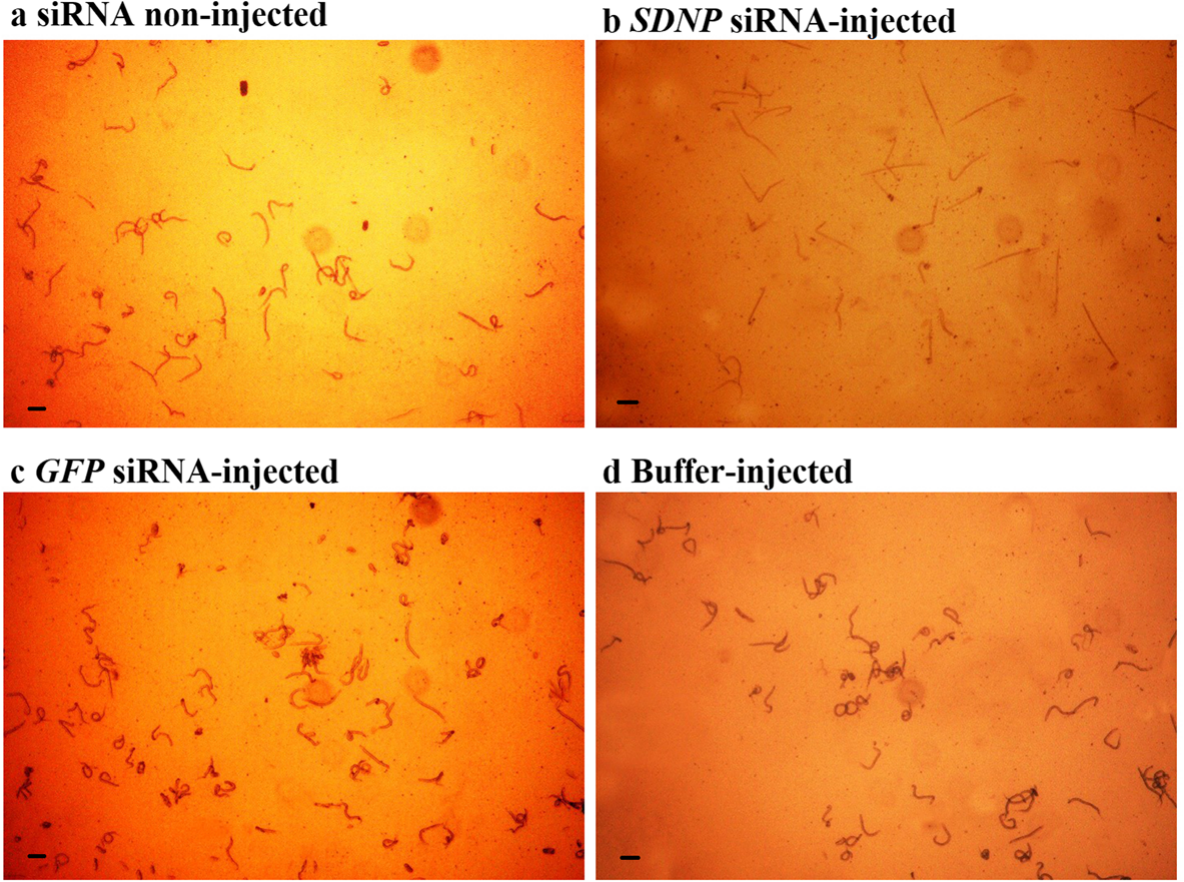
Motility comparison of larvae at 14 dpi in, **a** non-injected, **b** *SDNP* siRNA-injected, **c** *GFP* siRNA-injected, **d** buffer-injected mosquitoes (Magnification 40×) *Scale-bars*: **a**-**d**, 400 μm

**Fig. 6:**
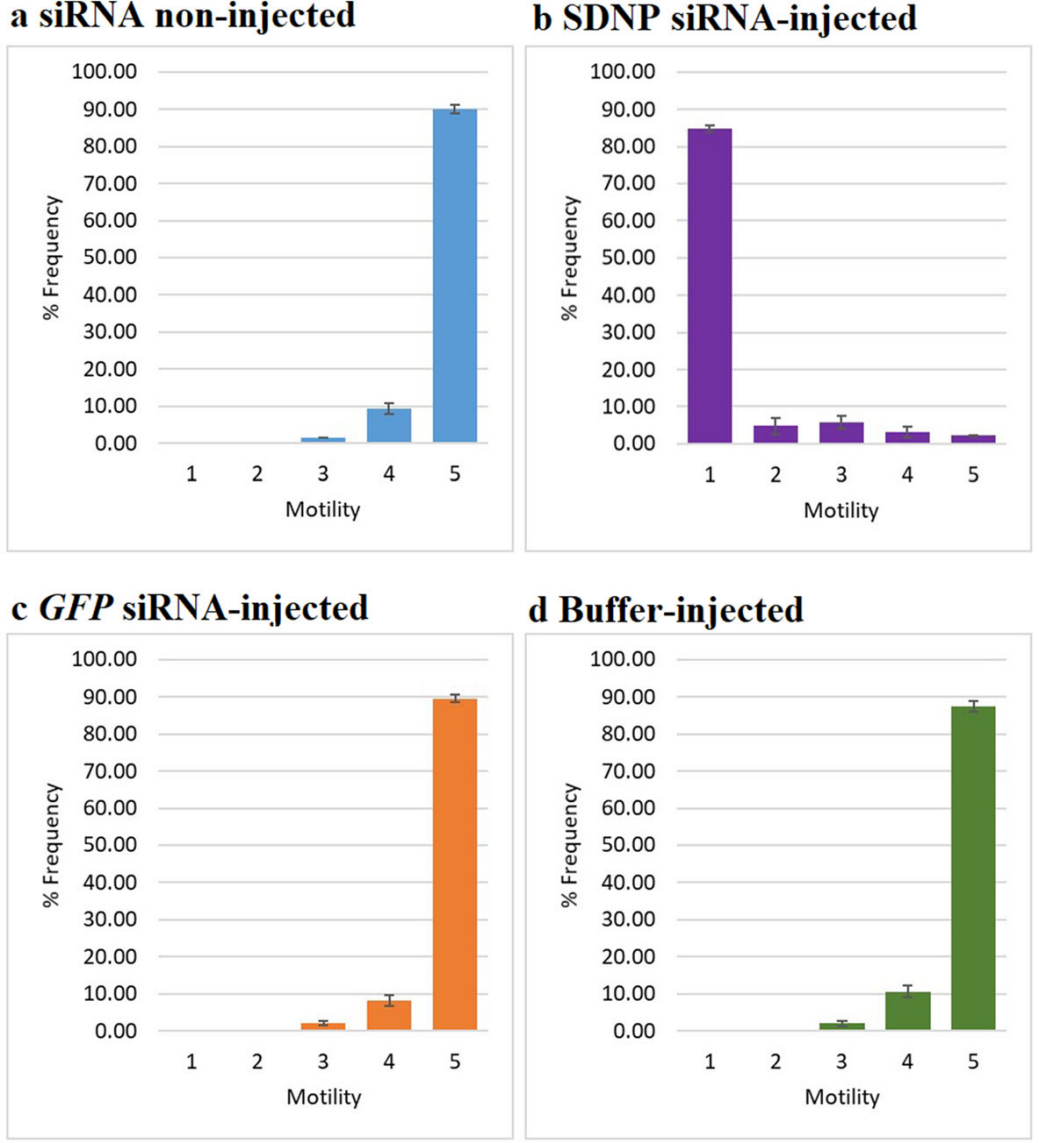
Frequency distribution for motility of *S. digitata* larvae at 14 dpi in, **a** siRNA non-injected, **b** *SDNP* siRNA-injected, **c** *GFP* siRNA-injected and **d** buffer-injected mosquitoes. The scoring scheme was: 1, immobile or dead; 2, compromised motility; 3, partial movement of the body; 4, slightly reduced motion in the body; 5, all parts of the body are vigorously twisting. Values in between 5 and 1 in the scale were assigned according to the decreasing motility blindly by an independent evaluator. Five replicates were performed for each experiment. Bars represent mean ± SD

**Fig. 7:**
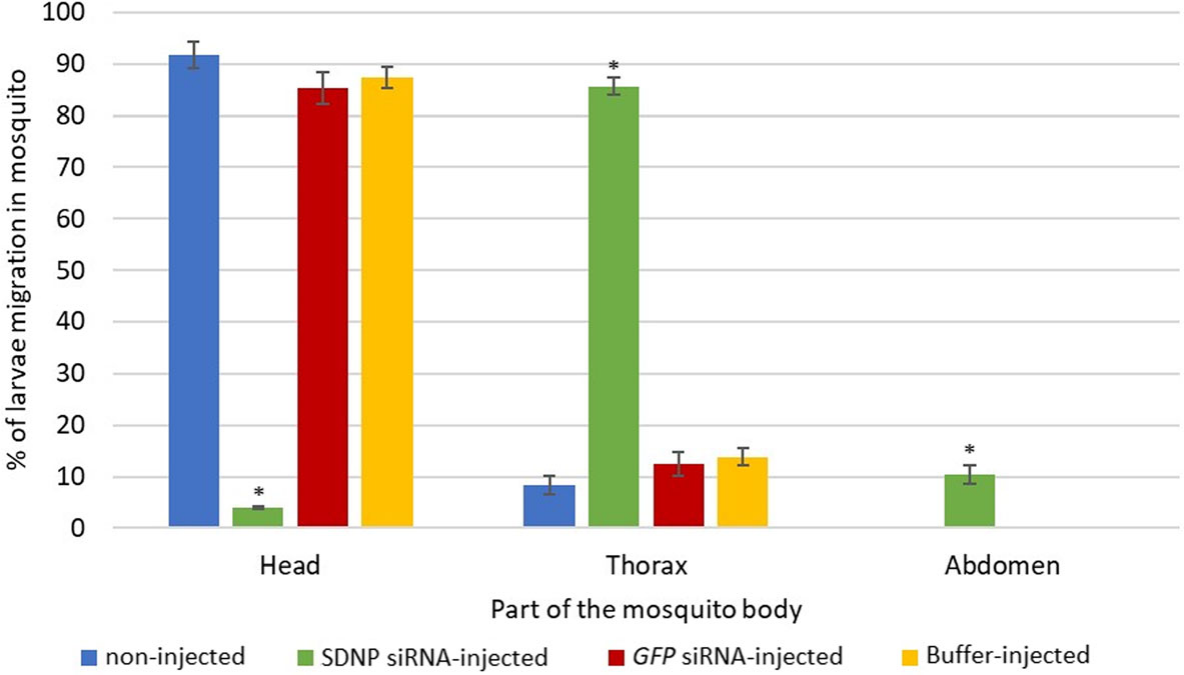
Frequency distribution of *S. digitata* larvae in *C. quinquefasciatus* head, thorax, or abdomen. Migration of the larvae was monitored at 14 dpi in non-injected, *SDNP* siRNA-injected, *GFP* siRNA-injected and buffer-injected mosquitoes. Mosquitoes were dissected into head, thorax and abdomen and larvae in each part was counted. Five replicates were performed for each experiment. Bars represent mean ± SD. Statistical analysis was performed using Student’s t-test (*P* < 0.05). Significant differences at *P* < 0.05 are indicated by *

**Fig. 8:**
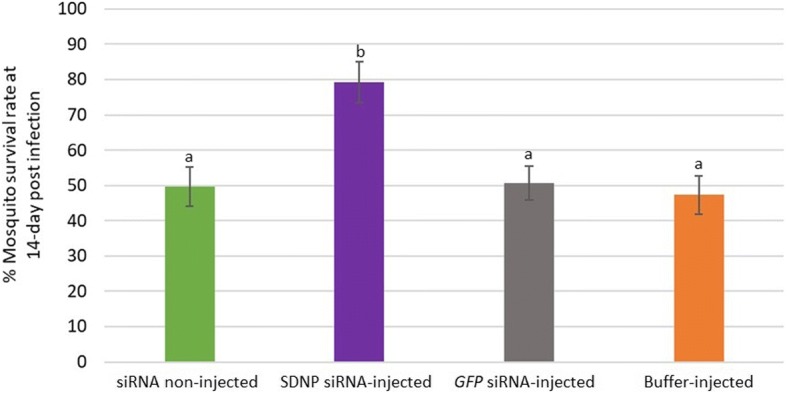
Mosquito survival rate. Survivability of the non-injected, *SDNP* siRNA-injected, *GFP* siRNA-injected and buffer-injected mosquitoes at 14 dpi was measured. Five replicates were performed for each experiment. Bars represent mean ± SD. Significant differences at *P* < 0.05 are indicated with different lowercase letters

In the motility changes monitored at 14 dpi of the control worms and worms from *SDNP* siRNA-injected mosquitoes, the former displayed a vigorously convoluting body movement (Fig. 5a, c, d) of 4 and 5 in the motility scale (Fig. 6a, c, d) while the *SDNP* siRNA-treated showed a significantly depressed body movement, feeble body twitching, and total body paralysis (Figs. 5b, 6b). Also, the worms from *SDNP* siRNA-injected worms and monitored at 14 dpi displayed irregular migration patterns in the mosquitoes compared to the controls (Fig. 7). Worms from the control mosquitoes were predominantly found in the head (non-injected: ~91%; GFP siRNA-injected: ~85%; buffer-injected: ~87%) of the mosquito, while *SDNP*-suppressed worms were mainly found either in the thorax (~85%) or abdomen (~10%) indicating that motility irregularities of larvae are associated with the migration within the mosquito. It has been previously revealed that *SDNP* had homology with the inter-domain linkers of the muscle-specific twitchin kinase of *C. elegans* and was expressed in the longitudinal muscles of the adult worm [24]. Hence, the downregulation of the *SDNP* could affect the muscle movement of the developing worm, which in turn reduces the migration of the parasite in the mosquito. The increased mosquito survival rate was seen in the *SDNP* siRNA-injected mosquito group compared to the control groups (Fig. 8). This observation suggests that the compromised motility and development of the larvae induced by the *SDNP* siRNA treatment could reduce the parasitic burden on the mosquito, thereby increasing its survival.

## Conclusions

In this study, we demonstrated the successful usage of the RNAi approach to turn off the expression of *SDNP* in the larval stages of *S. digitata* in their natural milieu, *C. quinquefasciatus*, and the possibility of using this host as an *in vivo* culture platform in the absence of proper culture conditions to study the biological functions of the genes that the *S. digitata* genome encodes*.* Further, we showed that the specific silencing of *SDNP* is associated with developmental deformities and motility changes in the developing larvae of *S. digitata*, in addition to the reduction of migration of the larvae to the head of the mosquito. Therefore, by taking the outcomes of present and our previous studies into consideration, it can be concluded that *SDNP* plays a vital role in the movement and development of larvae. Hence, it can be suggested that the *SDNP* may be utilised as a candidate anthelminthic drug target.

## Abbreviations

RNAi: RNA interference
SDNP: Setaria digitata novel protein
qPCR: Quantitative real-time polymerase chain reaction
siRNA: Small interfering RNA
dsRNA: Double stranded RNA
MPS: Mosquito physiological saline
MF: Microfilariae
hpi: Hour post infection
dpi: Days post-infection
GFP: Green fluorescent protein.
